# Tetra­kis(1*H*-imidazole-κ*N* ^3^)(2-phenyl­propanedioato-κ^2^ *O* ^1^,*O* ^3^)nickel(II)

**DOI:** 10.1107/S1600536810041577

**Published:** 2010-10-23

**Authors:** Kou-Lin Zhang, Guo-Wang Diao, Seik Weng Ng

**Affiliations:** a College of Chemistry and Chemical Engineering, Yangzhou University, Yangzhou 225002, People’s Republic of China; b Department of Chemistry, University of Malaya, 50603 Kuala Lumpur, Malaysia

## Abstract

In the title complex, [Ni(C_9_H_6_O_4_)(C_3_H_4_N_2_)_4_], the Ni^II^ ion is *O*,*O*′-chelated by the phenyl­malonato ligand and coordinated by four imidazole ligands in a slightly distorted octa­hedral geometry. In the crystal structure, symmetry-related mol­ecules are linked by N—H⋯O hydrogen bonds, generating a three-dimensional network.

## Related literature

For the cobalt(II) analog, see: Zhang *et al.* (2007 ▶).
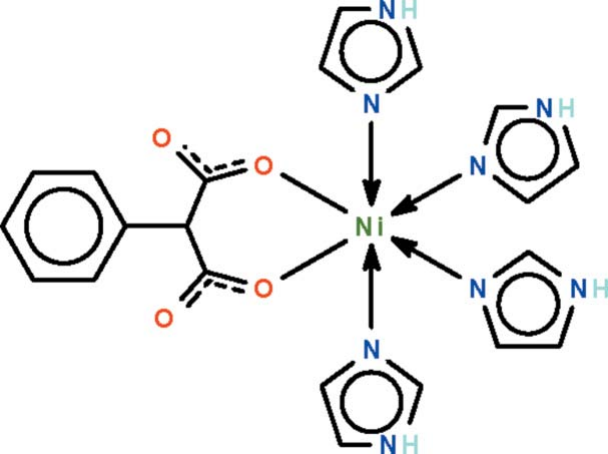


## Experimental

### 

#### Crystal data

[Ni(C_9_H_6_O_4_)(C_3_H_4_N_2_)_4_]
*M* *_r_* = 509.18
Orthorhombic, 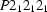

*a* = 8.5358 (8) Å
*b* = 13.3148 (12) Å
*c* = 20.6996 (19) Å
*V* = 2352.6 (4) Å^3^
*Z* = 4
Mo *K*α radiation
μ = 0.87 mm^−1^
*T* = 293 K
0.25 × 0.20 × 0.15 mm

#### Data collection

Bruker SMART APEX diffractometer
Absorption correction: multi-scan (*SADABS*; Sheldrick, 1996 ▶) *T*_min_ = 0.812, *T*_max_ = 0.881
15962 measured reflections
5516 independent reflections
4013 reflections with *I* > 2σ(*I*)
*R*_int_ = 0.051

#### Refinement

*R*[*F* ^2^ > 2σ(*F* ^2^)] = 0.041
*wR*(*F* ^2^) = 0.086
*S* = 0.98
5516 reflections
307 parameters
H-atom parameters constrained
Δρ_max_ = 0.41 e Å^−3^
Δρ_min_ = −0.31 e Å^−3^
Absolute structure: Flack (1983 ▶), 2344 Friedel pairs
Flack parameter: 0.082 (13)

### 

Data collection: *APEX2* (Bruker, 2005 ▶); cell refinement: *SAINT* (Bruker, 2005 ▶); data reduction: *SAINT*; program(s) used to solve structure: *SHELXS97* (Sheldrick, 2008 ▶); program(s) used to refine structure: *SHELXL97* (Sheldrick, 2008 ▶); molecular graphics: *X-SEED* (Barbour, 2001 ▶); software used to prepare material for publication: *publCIF* (Westrip, 2010 ▶).

## Supplementary Material

Crystal structure: contains datablocks global, I. DOI: 10.1107/S1600536810041577/lh5153sup1.cif

Structure factors: contains datablocks I. DOI: 10.1107/S1600536810041577/lh5153Isup2.hkl

Additional supplementary materials:  crystallographic information; 3D view; checkCIF report

## Figures and Tables

**Table 1:** Hydrogen-bond geometry (Å, °)

*D*—H⋯*A*	*D*—H	H⋯*A*	*D*⋯*A*	*D*—H⋯*A*
N2—H2⋯O3^i^	0.88	2.06	2.943 (3)	176
N4—H4⋯O1^ii^	0.88	1.97	2.839 (3)	168
N6—H6⋯O2^iii^	0.88	1.90	2.774 (3)	172
N8—H8⋯O4^iv^	0.88	1.85	2.718 (3)	169

Symmetry codes: (i) 
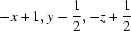
; (ii) 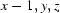; (iii) 
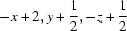
; (iv) 
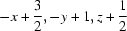
.

**Fig. 1.**
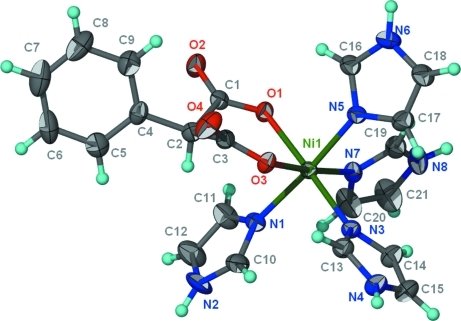
Thermal ellipsoid plot (Barbour, 2001) of Ni(C3H4N2)4(C9H6O4) at the 50% probability level; hydrogen atoms are drawn as spheres of arbitrary radius.

## supplementary crystallographic information

### Comment

We have previously reported the crystal structure of the tetrakis(imidazole) adduct of cobalt(II) phenylmalonate. The structure features the carboxylate-chelated cobalt(II) atom bonded to four *N*-heterocycles; each of these has a nitrogen-donor site that enables the octahedrally coordinated mononuclear molecule to connect with each other to form a three-dimensional network (Zhang *et al.*, 2007). The nickel analog (Fig. 1) is isostructural, the two compounds crystallizing with similar unit cell dimensions.

### Experimental

Imidazole (0.339 g. 0.56 mmol) was dissolved in methanol (10 ml) and to the solution was added nickel nitrate hexahydrate (0.480 g, 1.65 mmol) dissolved in water (6 ml). To the clear soluiton was added phenylmalonic acid (0.300 g, 1.65 mmol) and sodium hydroxide (0.120 g, 3.30 mmol) dissolved in water (10 ml). The filtered solution was set aside for the growth of green crystals over several days; yield 50%. CH&N elemental analysis. Calc. for C_21_H_22_N_8_NiO_4_: C 49.53, H 4.36, N 22.00%. Found: C 49.61, H 4.49, N 21.91%.

### Refinement

Carbon-bound H-atoms were placed in calculated positions (C–H 0.93–0.98 Å) and were included in the refinement in the riding model approximation, with *U*_iso_(H) set to 1.2*U*_eq_(C).

The imidazolium H-atoms were similarly treated (N–H 0.88 Å; *U*_iso_(H) 1.2*U*_eq_(N)).

The crystal has several voids but these are too small (20Å^3^) to accomodate a solvent molecule.

### Crystal data

**Table:**

[Ni(C_9_H_6_O_4_)(C_3_H_4_N_2_)_4_]	*F*(000) = 1056
*M**_r_* = 509.18	*D*_x_ = 1.438 Mg m^−^^3^
Orthorhombic, *P*2_1_2_1_2_1_	Mo *K*α radiation, λ = 0.71073 Å
Hall symbol: P 2ac 2ab	Cell parameters from 2481 reflections
*a* = 8.5358 (8) Å	θ = 2.6–21.7°
*b* = 13.3148 (12) Å	µ = 0.87 mm^−^^1^
*c* = 20.6996 (19) Å	*T* = 293 K
*V* = 2352.6 (4) Å^3^	Prism, green
*Z* = 4	0.25 × 0.20 × 0.15 mm

### Data collection

**Table:**

Bruker SMART APEX diffractometer	5516 independent reflections
Radiation source: fine-focus sealed tube	4013 reflections with *I* > 2σ(*I*)
graphite	*R*_int_ = 0.051
ω scans	θ_max_ = 27.8°, θ_min_ = 1.8°
Absorption correction: multi-scan (*SADABS*; Sheldrick, 1996)	*h* = −11→11
*T*_min_ = 0.812, *T*_max_ = 0.881	*k* = −17→17
15962 measured reflections	*l* = −19→27

### Refinement

**Table:**

Refinement on *F*^2^	Secondary atom site location: difference Fourier map
Least-squares matrix: full	Hydrogen site location: inferred from neighbouring sites
*R*[*F*^2^ > 2σ(*F*^2^)] = 0.041	H-atom parameters constrained
*wR*(*F*^2^) = 0.086	*w* = 1/[σ^2^(*F*_o_^2^) + (0.0326*P*)^2^] where *P* = (*F*_o_^2^ + 2*F*_c_^2^)/3
*S* = 0.98	(Δ/σ)_max_ = 0.001
5516 reflections	Δρ_max_ = 0.41 e Å^−^^3^
307 parameters	Δρ_min_ = −0.31 e Å^−^^3^
0 restraints	Absolute structure: Flack (1983), 2344 Friedel pairs
Primary atom site location: structure-invariant direct methods	Flack parameter: 0.082 (13)

### Fractional atomic coordinates and isotropic or equivalent isotropic displacement parameters (Å^2^)

**Table:**

	*x*	*y*	*z*	*U*_iso_*/*U*_eq_	
Ni1	0.66167 (4)	0.40568 (3)	0.336577 (18)	0.02636 (10)	
O1	0.8851 (2)	0.40575 (17)	0.29819 (9)	0.0324 (4)	
O2	1.0679 (2)	0.37546 (16)	0.22649 (12)	0.0433 (6)	
O3	0.5823 (2)	0.41866 (15)	0.24132 (9)	0.0323 (5)	
O4	0.6203 (3)	0.43579 (17)	0.13674 (11)	0.0515 (7)	
N1	0.6652 (3)	0.24683 (16)	0.33088 (13)	0.0338 (6)	
N2	0.5969 (3)	0.0939 (2)	0.30389 (16)	0.0597 (9)	
H2	0.5388	0.0432	0.2908	0.072*	
N3	0.4310 (2)	0.4058 (2)	0.37002 (11)	0.0301 (5)	
N4	0.1739 (3)	0.4091 (2)	0.36620 (12)	0.0388 (6)	
H4	0.0782	0.4110	0.3504	0.047*	
N5	0.6703 (3)	0.56328 (16)	0.33579 (12)	0.0312 (5)	
N6	0.7570 (3)	0.7169 (2)	0.32201 (14)	0.0424 (8)	
H6	0.8180	0.7667	0.3098	0.051*	
N7	0.7489 (3)	0.4034 (2)	0.43160 (11)	0.0324 (6)	
N8	0.8143 (4)	0.4576 (2)	0.52810 (14)	0.0500 (8)	
H8	0.8359	0.4991	0.5599	0.060*	
C1	0.9291 (3)	0.3761 (2)	0.24304 (17)	0.0300 (7)	
C2	0.8045 (3)	0.3333 (2)	0.19607 (15)	0.0343 (8)	
H2A	0.7662	0.2713	0.2161	0.041*	
C3	0.6602 (3)	0.4033 (2)	0.19068 (14)	0.0315 (6)	
C4	0.8732 (3)	0.3022 (2)	0.13164 (16)	0.0361 (8)	
C5	0.8607 (4)	0.2032 (3)	0.11128 (17)	0.0468 (9)	
H5	0.8055	0.1569	0.1360	0.056*	
C6	0.9301 (5)	0.1738 (3)	0.0545 (2)	0.0635 (12)	
H6A	0.9202	0.1075	0.0410	0.076*	
C7	1.0126 (5)	0.2392 (4)	0.0179 (2)	0.0683 (13)	
H7	1.0602	0.2173	−0.0199	0.082*	
C8	1.0264 (5)	0.3368 (3)	0.0363 (2)	0.0643 (12)	
H8A	1.0831	0.3817	0.0110	0.077*	
C9	0.9556 (4)	0.3689 (3)	0.09264 (17)	0.0471 (9)	
H9	0.9632	0.4360	0.1047	0.057*	
C10	0.5503 (4)	0.1886 (2)	0.31110 (17)	0.0450 (9)	
H10	0.4488	0.2108	0.3031	0.054*	
C11	0.7919 (4)	0.1839 (2)	0.3360 (2)	0.0493 (9)	
H11	0.8920	0.2036	0.3484	0.059*	
C12	0.7502 (4)	0.0899 (3)	0.3204 (2)	0.0626 (12)	
H12	0.8139	0.0333	0.3208	0.075*	
C13	0.3064 (3)	0.4087 (2)	0.33245 (16)	0.0361 (7)	
H13	0.3106	0.4102	0.2876	0.043*	
C14	0.3722 (3)	0.4050 (3)	0.43166 (15)	0.0391 (7)	
H14	0.4318	0.4040	0.4693	0.047*	
C15	0.2137 (4)	0.4060 (3)	0.42918 (17)	0.0446 (8)	
H15	0.1454	0.4048	0.4642	0.054*	
C16	0.7835 (4)	0.6195 (2)	0.31334 (16)	0.0383 (8)	
H16	0.8728	0.5941	0.2935	0.046*	
C17	0.5650 (4)	0.6304 (2)	0.36077 (17)	0.0427 (9)	
H17	0.4705	0.6132	0.3802	0.051*	
C18	0.6178 (4)	0.7249 (2)	0.35329 (17)	0.0426 (9)	
H18	0.5690	0.7837	0.3668	0.051*	
C19	0.7768 (4)	0.4830 (3)	0.46802 (16)	0.0392 (8)	
H19	0.7709	0.5489	0.4533	0.047*	
C20	0.7710 (5)	0.3241 (3)	0.47194 (18)	0.0585 (12)	
H20	0.7590	0.2571	0.4604	0.070*	
C21	0.8133 (6)	0.3571 (3)	0.53140 (18)	0.0682 (13)	
H21	0.8368	0.3179	0.5673	0.082*	

### Atomic displacement parameters (Å^2^)

**Table:**

	*U*^11^	*U*^22^	*U*^33^	*U*^12^	*U*^13^	*U*^23^
Ni1	0.02413 (16)	0.02630 (16)	0.02864 (19)	−0.00065 (17)	−0.00168 (18)	−0.00153 (19)
O1	0.0262 (10)	0.0397 (11)	0.0312 (11)	−0.0002 (10)	0.0005 (9)	−0.0080 (11)
O2	0.0255 (11)	0.0565 (15)	0.0479 (15)	0.0019 (10)	0.0034 (11)	−0.0166 (12)
O3	0.0296 (10)	0.0422 (13)	0.0252 (11)	0.0041 (10)	0.0009 (9)	−0.0002 (10)
O4	0.0486 (14)	0.0700 (17)	0.0359 (14)	0.0183 (12)	0.0071 (12)	0.0217 (12)
N1	0.0380 (13)	0.0312 (13)	0.0322 (15)	−0.0052 (12)	−0.0028 (18)	0.0004 (11)
N2	0.0581 (18)	0.0281 (14)	0.093 (3)	−0.0143 (16)	−0.0013 (17)	−0.0146 (18)
N3	0.0258 (11)	0.0332 (12)	0.0313 (14)	−0.0023 (13)	−0.0017 (11)	−0.0005 (14)
N4	0.0232 (11)	0.0497 (15)	0.0436 (15)	0.0030 (15)	−0.0003 (13)	0.0011 (15)
N5	0.0278 (12)	0.0303 (12)	0.0356 (14)	−0.0031 (10)	0.0006 (17)	−0.0003 (11)
N6	0.0387 (15)	0.0343 (15)	0.054 (2)	−0.0088 (13)	−0.0008 (14)	0.0059 (15)
N7	0.0331 (12)	0.0349 (13)	0.0291 (14)	0.0015 (13)	−0.0029 (11)	−0.0033 (15)
N8	0.064 (2)	0.053 (2)	0.0330 (18)	0.0026 (18)	−0.0074 (17)	−0.0125 (15)
C1	0.0243 (15)	0.0255 (16)	0.040 (2)	0.0061 (12)	−0.0016 (14)	−0.0010 (13)
C2	0.0326 (18)	0.0358 (17)	0.0346 (18)	−0.0004 (14)	0.0001 (15)	0.0012 (15)
C3	0.0283 (13)	0.0343 (14)	0.0319 (15)	−0.0006 (18)	0.0009 (14)	−0.0023 (15)
C4	0.0273 (16)	0.049 (2)	0.0322 (17)	0.0077 (15)	−0.0009 (14)	−0.0051 (16)
C5	0.0362 (18)	0.051 (2)	0.053 (2)	0.0041 (18)	−0.0058 (18)	−0.0053 (18)
C6	0.064 (3)	0.069 (3)	0.057 (3)	0.018 (2)	−0.007 (2)	−0.031 (2)
C7	0.075 (3)	0.088 (3)	0.042 (3)	0.032 (3)	−0.001 (2)	−0.013 (2)
C8	0.060 (3)	0.088 (3)	0.045 (3)	0.018 (2)	0.015 (2)	0.015 (2)
C9	0.052 (2)	0.048 (2)	0.042 (2)	0.0090 (17)	0.0023 (19)	0.0043 (17)
C10	0.0379 (18)	0.0350 (18)	0.062 (3)	−0.0055 (15)	0.0071 (17)	−0.0004 (17)
C11	0.0431 (19)	0.0379 (18)	0.067 (3)	0.0085 (14)	−0.018 (2)	−0.004 (2)
C12	0.056 (2)	0.0342 (19)	0.098 (4)	0.006 (2)	−0.009 (2)	−0.003 (3)
C13	0.0293 (14)	0.0416 (16)	0.0374 (17)	−0.0031 (14)	−0.0022 (14)	−0.0015 (19)
C14	0.0350 (16)	0.0509 (18)	0.0313 (17)	0.0019 (18)	−0.0008 (14)	0.0026 (18)
C15	0.0366 (16)	0.056 (2)	0.041 (2)	0.0009 (19)	0.0089 (15)	0.006 (2)
C16	0.0365 (17)	0.0314 (19)	0.047 (2)	0.0011 (13)	0.0027 (15)	−0.0013 (15)
C17	0.0347 (17)	0.0400 (18)	0.053 (2)	0.0016 (14)	0.0081 (17)	0.0003 (16)
C18	0.0430 (19)	0.0261 (16)	0.059 (3)	0.0068 (14)	0.0016 (17)	0.0031 (15)
C19	0.0401 (19)	0.044 (2)	0.034 (2)	−0.0004 (16)	−0.0038 (16)	−0.0028 (17)
C20	0.102 (3)	0.038 (2)	0.036 (2)	0.003 (2)	−0.020 (2)	0.0050 (17)
C21	0.112 (4)	0.057 (3)	0.036 (2)	0.006 (3)	−0.025 (3)	0.0050 (19)

### Geometric parameters (Å, °)

**Table:**

Ni1—O1	2.0662 (18)	C2—C4	1.515 (4)
Ni1—N3	2.087 (2)	C2—C3	1.549 (4)
Ni1—O3	2.092 (2)	C2—H2A	0.9800
Ni1—N5	2.100 (2)	C4—C5	1.388 (5)
Ni1—N7	2.103 (2)	C4—C9	1.391 (4)
Ni1—N1	2.118 (2)	C5—C6	1.372 (5)
O1—C1	1.265 (4)	C5—H5	0.9300
O2—C1	1.234 (3)	C6—C7	1.352 (6)
O3—C3	1.258 (3)	C6—H6A	0.9300
O4—C3	1.245 (3)	C7—C8	1.359 (5)
N1—C10	1.316 (4)	C7—H7	0.9300
N1—C11	1.373 (4)	C8—C9	1.382 (5)
N2—C10	1.330 (4)	C8—H8A	0.9300
N2—C12	1.353 (4)	C9—H9	0.9300
N2—H2	0.8800	C10—H10	0.9300
N3—C13	1.318 (3)	C11—C12	1.340 (5)
N3—C14	1.371 (4)	C11—H11	0.9300
N4—C13	1.329 (3)	C12—H12	0.9300
N4—C15	1.348 (4)	C13—H13	0.9300
N4—H4	0.8800	C14—C15	1.355 (4)
N5—C16	1.308 (4)	C14—H14	0.9300
N5—C17	1.369 (4)	C15—H15	0.9300
N6—C16	1.329 (4)	C16—H16	0.9300
N6—C18	1.357 (4)	C17—C18	1.345 (4)
N6—H6	0.8800	C17—H17	0.9300
N7—C19	1.323 (4)	C18—H18	0.9300
N7—C20	1.359 (4)	C19—H19	0.9300
N8—C19	1.328 (4)	C20—C21	1.356 (5)
N8—C21	1.339 (4)	C20—H20	0.9300
N8—H8	0.8800	C21—H21	0.9300
C1—C2	1.550 (4)		
			
O1—Ni1—N3	176.75 (9)	C5—C4—C2	119.8 (3)
O1—Ni1—O3	86.37 (7)	C9—C4—C2	122.2 (3)
N3—Ni1—O3	90.39 (8)	C6—C5—C4	119.9 (4)
O1—Ni1—N5	87.95 (9)	C6—C5—H5	120.1
N3—Ni1—N5	91.98 (10)	C4—C5—H5	120.1
O3—Ni1—N5	85.50 (9)	C7—C6—C5	121.4 (4)
O1—Ni1—N7	91.88 (8)	C7—C6—H6A	119.3
N3—Ni1—N7	91.36 (9)	C5—C6—H6A	119.3
O3—Ni1—N7	175.72 (9)	C6—C7—C8	120.3 (4)
N5—Ni1—N7	90.54 (10)	C6—C7—H7	119.9
O1—Ni1—N1	88.05 (10)	C8—C7—H7	119.9
N3—Ni1—N1	91.88 (10)	C7—C8—C9	119.6 (4)
O3—Ni1—N1	91.98 (9)	C7—C8—H8A	120.2
N5—Ni1—N1	175.40 (10)	C9—C8—H8A	120.2
N7—Ni1—N1	91.87 (10)	C8—C9—C4	120.9 (4)
C1—O1—Ni1	128.36 (18)	C8—C9—H9	119.5
C3—O3—Ni1	126.92 (18)	C4—C9—H9	119.5
C10—N1—C11	104.6 (2)	N1—C10—N2	111.8 (3)
C10—N1—Ni1	126.6 (2)	N1—C10—H10	124.1
C11—N1—Ni1	128.0 (2)	N2—C10—H10	124.1
C10—N2—C12	107.3 (3)	C12—C11—N1	110.0 (3)
C10—N2—H2	126.3	C12—C11—H11	125.0
C12—N2—H2	126.3	N1—C11—H11	125.0
C13—N3—C14	104.7 (2)	C11—C12—N2	106.3 (4)
C13—N3—Ni1	124.44 (19)	C11—C12—H12	126.8
C14—N3—Ni1	130.84 (18)	N2—C12—H12	126.8
C13—N4—C15	107.1 (2)	N3—C13—N4	112.1 (3)
C13—N4—H4	126.5	N3—C13—H13	123.9
C15—N4—H4	126.5	N4—C13—H13	123.9
C16—N5—C17	104.2 (3)	C15—C14—N3	109.3 (3)
C16—N5—Ni1	126.9 (2)	C15—C14—H14	125.4
C17—N5—Ni1	128.8 (2)	N3—C14—H14	125.4
C16—N6—C18	106.8 (3)	N4—C15—C14	106.8 (3)
C16—N6—H6	126.6	N4—C15—H15	126.6
C18—N6—H6	126.6	C14—C15—H15	126.6
C19—N7—C20	104.3 (3)	N5—C16—N6	112.7 (3)
C19—N7—Ni1	125.8 (2)	N5—C16—H16	123.7
C20—N7—Ni1	129.4 (2)	N6—C16—H16	123.7
C19—N8—C21	107.5 (3)	C18—C17—N5	110.3 (3)
C19—N8—H8	126.2	C18—C17—H17	124.8
C21—N8—H8	126.2	N5—C17—H17	124.8
O2—C1—O1	122.5 (3)	C17—C18—N6	106.0 (3)
O2—C1—C2	118.9 (3)	C17—C18—H18	127.0
O1—C1—C2	118.5 (3)	N6—C18—H18	127.0
C4—C2—C3	114.1 (2)	N7—C19—N8	111.9 (3)
C4—C2—C1	112.8 (2)	N7—C19—H19	124.1
C3—C2—C1	111.7 (2)	N8—C19—H19	124.1
C4—C2—H2A	105.8	C21—C20—N7	110.1 (3)
C3—C2—H2A	105.8	C21—C20—H20	125.0
C1—C2—H2A	105.8	N7—C20—H20	125.0
O4—C3—O3	123.1 (3)	N8—C21—C20	106.2 (4)
O4—C3—C2	119.4 (3)	N8—C21—H21	126.9
O3—C3—C2	117.2 (3)	C20—C21—H21	126.9
C5—C4—C9	118.0 (3)		
			
O3—Ni1—O1—C1	−28.3 (3)	Ni1—O3—C3—O4	−161.4 (2)
N5—Ni1—O1—C1	−113.9 (3)	Ni1—O3—C3—C2	23.8 (4)
N7—Ni1—O1—C1	155.7 (3)	C4—C2—C3—O4	−6.3 (4)
N1—Ni1—O1—C1	63.9 (3)	C1—C2—C3—O4	123.0 (3)
O1—Ni1—O3—C3	15.1 (2)	C4—C2—C3—O3	168.7 (3)
N3—Ni1—O3—C3	−164.7 (2)	C1—C2—C3—O3	−61.9 (4)
N5—Ni1—O3—C3	103.4 (2)	C3—C2—C4—C5	−111.2 (3)
N1—Ni1—O3—C3	−72.8 (2)	C1—C2—C4—C5	120.0 (3)
O1—Ni1—N1—C10	−137.2 (3)	C3—C2—C4—C9	71.8 (4)
N3—Ni1—N1—C10	39.6 (3)	C1—C2—C4—C9	−57.1 (4)
O3—Ni1—N1—C10	−50.9 (3)	C9—C4—C5—C6	0.6 (5)
N7—Ni1—N1—C10	131.0 (3)	C2—C4—C5—C6	−176.6 (3)
O1—Ni1—N1—C11	30.7 (3)	C4—C5—C6—C7	0.8 (6)
N3—Ni1—N1—C11	−152.6 (3)	C5—C6—C7—C8	−1.1 (7)
O3—Ni1—N1—C11	117.0 (3)	C6—C7—C8—C9	0.1 (7)
N7—Ni1—N1—C11	−61.1 (3)	C7—C8—C9—C4	1.4 (6)
O3—Ni1—N3—C13	2.8 (3)	C5—C4—C9—C8	−1.7 (5)
N5—Ni1—N3—C13	88.3 (3)	C2—C4—C9—C8	175.4 (3)
N7—Ni1—N3—C13	178.9 (3)	C11—N1—C10—N2	0.3 (4)
N1—Ni1—N3—C13	−89.2 (3)	Ni1—N1—C10—N2	170.4 (2)
O3—Ni1—N3—C14	−175.9 (3)	C12—N2—C10—N1	0.5 (4)
N5—Ni1—N3—C14	−90.4 (3)	C10—N1—C11—C12	−1.0 (4)
N7—Ni1—N3—C14	0.2 (3)	Ni1—N1—C11—C12	−171.0 (3)
N1—Ni1—N3—C14	92.1 (3)	N1—C11—C12—N2	1.3 (5)
O1—Ni1—N5—C16	3.4 (3)	C10—N2—C12—C11	−1.1 (5)
N3—Ni1—N5—C16	−173.4 (3)	C14—N3—C13—N4	−0.4 (4)
O3—Ni1—N5—C16	−83.1 (3)	Ni1—N3—C13—N4	−179.4 (2)
N7—Ni1—N5—C16	95.3 (3)	C15—N4—C13—N3	−0.2 (4)
O1—Ni1—N5—C17	−173.2 (3)	C13—N3—C14—C15	0.8 (4)
N3—Ni1—N5—C17	10.1 (3)	Ni1—N3—C14—C15	179.7 (2)
O3—Ni1—N5—C17	100.3 (3)	C13—N4—C15—C14	0.7 (4)
N7—Ni1—N5—C17	−81.3 (3)	N3—C14—C15—N4	−1.0 (4)
O1—Ni1—N7—C19	92.3 (2)	C17—N5—C16—N6	−0.3 (4)
N3—Ni1—N7—C19	−87.6 (3)	Ni1—N5—C16—N6	−177.6 (2)
N5—Ni1—N7—C19	4.4 (3)	C18—N6—C16—N5	1.1 (4)
N1—Ni1—N7—C19	−179.5 (3)	C16—N5—C17—C18	−0.5 (4)
O1—Ni1—N7—C20	−96.5 (3)	Ni1—N5—C17—C18	176.7 (2)
N3—Ni1—N7—C20	83.5 (3)	N5—C17—C18—N6	1.2 (4)
N5—Ni1—N7—C20	175.5 (3)	C16—N6—C18—C17	−1.3 (4)
N1—Ni1—N7—C20	−8.4 (3)	C20—N7—C19—N8	−0.1 (4)
Ni1—O1—C1—O2	−176.5 (2)	Ni1—N7—C19—N8	172.8 (2)
Ni1—O1—C1—C2	−0.1 (4)	C21—N8—C19—N7	0.9 (5)
O2—C1—C2—C4	−4.3 (4)	C19—N7—C20—C21	−0.6 (5)
O1—C1—C2—C4	179.1 (3)	Ni1—N7—C20—C21	−173.2 (3)
O2—C1—C2—C3	−134.4 (3)	C19—N8—C21—C20	−1.2 (6)
O1—C1—C2—C3	49.0 (4)	N7—C20—C21—N8	1.2 (6)

### Hydrogen-bond geometry (Å, °)

**Table:**

*D*—H···*A*	*D*—H	H···*A*	*D*···*A*	*D*—H···*A*
N2—H2···O3^i^	0.88	2.06	2.943 (3)	176
N4—H4···O1^ii^	0.88	1.97	2.839 (3)	168
N6—H6···O2^iii^	0.88	1.90	2.774 (3)	172
N8—H8···O4^iv^	0.88	1.85	2.718 (3)	169

Symmetry codes: (i) −*x*+1, *y*−1/2, −*z*+1/2; (ii) *x*−1, *y*, *z*; (iii) −*x*+2, *y*+1/2, −*z*+1/2; (iv) −*x*+3/2, −*y*+1, *z*+1/2.
